# Cetacean Morbillivirus: Current Knowledge and Future Directions

**DOI:** 10.3390/v6125145

**Published:** 2014-12-22

**Authors:** Marie-Françoise Van Bressem, Pádraig J. Duignan, Ashley Banyard, Michelle Barbieri, Kathleen M Colegrove, Sylvain De Guise, Giovanni Di Guardo, Andrew Dobson, Mariano Domingo, Deborah Fauquier, Antonio Fernandez, Tracey Goldstein, Bryan Grenfell, Kátia R. Groch, Frances Gulland, Brenda A Jensen, Paul D Jepson, Ailsa Hall, Thijs Kuiken, Sandro Mazzariol, Sinead E Morris, Ole Nielsen, Juan A Raga, Teresa K Rowles, Jeremy Saliki, Eva Sierra, Nahiid Stephens, Brett Stone, Ikuko Tomo, Jianning Wang, Thomas Waltzek, James FX Wellehan

**Affiliations:** 1 Cetacean Conservation Medicine Group (CMED), Peruvian Centre for Cetacean Research (CEPEC), Pucusana, Lima 20, Peru; 2 Department of Ecosystem and Public Health, University of Calgary, Calgary, AL T2N 4Z6, Canada; E-Mail: ppjduign@ucalgary.ca; 3 Wildlife Zoonoses and Vector Borne Disease Research Group, Animal and Plant Health Agency (APHA), Weybridge, Surrey KT15 3NB, UK; E-Mail: ashley.banyard@apha.gsi.gov.uk; 4 The Marine Mammal Centre, Sausalito, CA 94965, USA; E-Mails: michelle.barbieri@noaa.gov (M.B.); gullandf@tmmc.org (F.G.); 5 Zoological Pathology Program, College of Veterinary Medicine, University of Illinois at Maywood, IL 60153 , USA; E-Mail: katie.colegrove@gmail.com; 6 Department of Pathobiology and Veterinary Science, and Connecticut Sea Grant College Program, University of Connecticut, Storrs, CT 06269, USA; E-Mail: sylvain.deguise@uconn.edu; 7 Faculty of Veterinary Medicine, University of Teramo, 64100 Teramo, Italy; E-Mail: gdiguardo@unite.it; 8 Department of Ecology and Evolutionary Biology, Princeton University, Princeton, NJ 08544, USA; E-Mails: dobson@princeton.edu (A.D.); grenfell@princeton.edu (B.G.); semorris@princeton.edu (S.E.M.); 9 Centre de Recerca en Sanitat Animal (CReSA), Autonomous University of Barcelona, Bellaterra, Barcelona 08193, Spain; E-Mail: Mariano.Domingo@cresa.uab.cat; 10 National Marine Fisheries Service, Marine Mammal Health and Stranding Response Program, Silver Spring, MD 20910, USA; E-Mails: deborah.fauquier@noaa.gov (D.F.); teri.rowles@noaa.gov (T.K.R.); 11 Department of Veterinary Pathology, Institute of Animal Health, Veterinary School, Universidad de Las Palmas de Gran Canaria, Las Palmas 35413, Spain; E-Mails: afernandez@dmor.ulpgc.es (A.F.); esierra@becarios.ulpgc.es (E.S.); 12 One Health Institute School of Veterinary Medicine University of California, Davis, CA 95616, USA; E-Mail: tgoldstein@ucdavis.edu; 13 Fogarty International Center, National Institutes of Health, Bethesda, MD 20892, USA; 14 Department of Pathology, School of Veterinary Medicine and Animal Sciences, University of São Paulo, São Paulo 05508-207, Brazil; E-Mail: katia.groch@gmail.com; 15 Instituto Baleia Jubarte (Humpback Whale Institute), Caravelas, Bahia 45900-000, Brazil; 16 Marine Mammal Commission, 4340 East-West Highway, Bethesda, MD 20814, USA; 17 Department of Natural Sciences, Hawai`i Pacific University, Kaneohe, HI 96744, USA; E-Mail: bjensen@hpu.edu; 18 Institute of Zoology, Regent’s Park, London NW1 4RY, UK; E-Mail: paul.jepson@ioz.ac.uk; 19 Sea Mammal Research Unit, Scottish Oceans Institute, University of St. Andrews, St. Andrews KY16 8LB, UK; E-Mail: ajh7@st-andrews.ac.uk; 20 Department of Viroscience, Erasmus MC, Rotterdam 3015 CN, The Netherlands; E-Mail: t.kuiken@erasmusmc.nl; 21 Department of Comparative Biomedicine and Food Science, University of Padua, Padua 35020, Italy; E-Mail: sandro.mazzariol@unipd.it; 22 Department of Fisheries and Oceans Canada, Central and Arctic Region, 501 University Crescent, Winnipeg, MB R3T 2N6 , Canada; E-Mail: ole.nielsen@dfo-mpo.gc.ca; 23 Marine Zoology Unit, Cavanilles Institute of Biodiversity and Evolutionary Biology, University of Valencia, Valencia 22085, Spain; E-Mail: raga@uv.es; 24 Athens Veterinary Diagnostic Laboratory, College of Veterinary Medicine, University of Georgia, Athens, GA GA 30602 , USA; E-Mail: jsaliki@uga.edu; 25 School of Veterinary and Life Sciences, Murdoch University, Perth 6150, Western Australia, Australia; E-Mail: n.stephens@murdoch.edu.au; 26 QML Vetnostics, Metroplex on Gateway, Murarrie, Queensland 4172, Australia; E-Mail: brett.stone@qml.com.au; 27 South Australian Museum, North Terrace, Adelaide 5000, South Australia, Australia; E-Mail: ikuko.tomo@samuseum.sa.gov.au; 28 Commonwealth Scientific and Industrial Research Organisation (CSIRO), East Geelong, Victoria 3220, Australia; E-Mail: Jianning.Wang@csiro.au; 29 Department of Infectious Diseases and Pathology, College of Veterinary Medicine, University of Florida, Gainesville, FL 32611, USA; E-Mail: tomwaltzek@gmail.com; 30 Department of Small Animal Clinical Sciences, College of Veterinary Medicine, University of Florida, Gainesville, FL 32611, USA; E-Mail: wellehanj@ufl.edu

**Keywords:** cetacean morbillivirus, epidemics, mass stranding, SLAM, phylogeny, pathogenesis, diagnosis, endemic infections

## Abstract

We review the molecular and epidemiological characteristics of cetacean morbillivirus (CeMV) and the diagnosis and pathogenesis of associated disease, with six different strains detected in cetaceans worldwide. CeMV has caused epidemics with high mortality in odontocetes in Europe, the USA and Australia. It represents a distinct species within the *Morbillivirus* genus. Although most CeMV strains are phylogenetically closely related, recent data indicate that morbilliviruses recovered from Indo-Pacific bottlenose dolphins (*Tursiops aduncus*), from Western Australia, and a Guiana dolphin (*Sotalia guianensis*), from Brazil, are divergent. The signaling lymphocyte activation molecule (SLAM) cell receptor for CeMV has been characterized in cetaceans. It shares higher amino acid identity with the ruminant SLAM than with the receptors of carnivores or humans, reflecting the evolutionary history of these mammalian taxa. In Delphinidae, three amino acid substitutions may result in a higher affinity for the virus. Infection is diagnosed by histology, immunohistochemistry, virus isolation, RT-PCR, and serology. Classical CeMV-associated lesions include bronchointerstitial pneumonia, encephalitis, syncytia, and lymphoid depletion associated with immunosuppression. Cetaceans that survive the acute disease may develop fatal secondary infections and chronic encephalitis. Endemically infected, gregarious odontocetes probably serve as reservoirs and vectors. Transmission likely occurs through the inhalation of aerosolized virus but mother to fetus transmission was also reported.

## 1. Introduction

**Figure 1:**
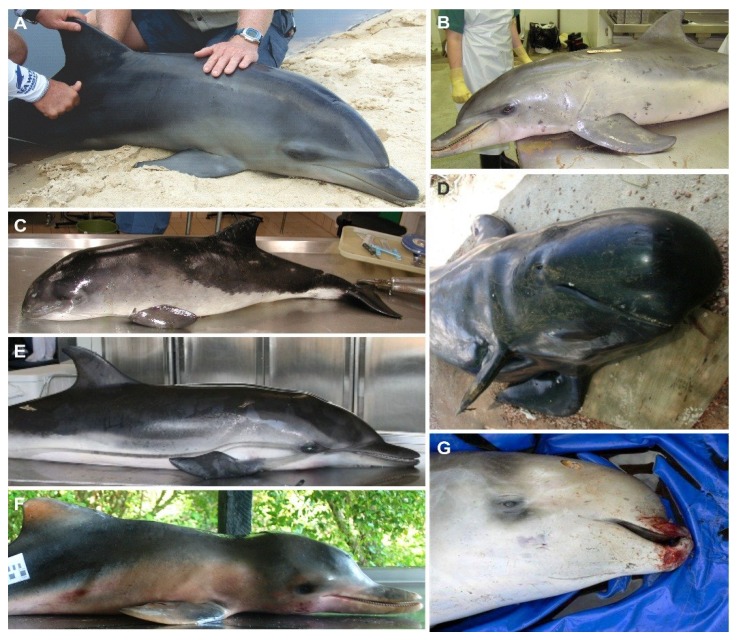
Cetacean species in which the six CeMV strains were isolated or detected by RT-PCR. (**A**) Common bottlenose dolphin (*Tursiops truncatus*), Fraser Island, Australia, 2010 (© E. Pearce); (**B**) Indo-Pacific bottlenose dolphin (*Tursiops aduncus*), Swan River, Perth, Australia, 2009 (© N. Stephens); (**C**) Harbour porpoise (*Phocoena phocoena*), Kent, UK, 2005 (© R. Deaville); (**D**) Long-finned pilot whale (*Globicephala melas*), Alicante, Spain, 2007 (© A.J. Raga); (**E**) Striped dolphin (*Stenella coeruleoalba*), Valencia, Spain, 2007 (© A.J. Raga); (**F**) Emaciated calf Guiana dolphin (*Sotalia guianensis*), Guriri, Espirito Santo, Brazil 2010 (© K. Groch); (**G**) Longman’s beaked whale (*Indopacetus pacificus*), Hawaii, US, March 2010 (© K. West, Hawaii Pacific University, NOAA Permit number 932-1905).

Cetacean morbillivirus (CeMV) is a recently described member of the genus *Morbillivirus*, subfamily *Paramyxovirinae,* family *Paramyxoviridae*, Order Mononegavirales, that includes three well characterized strains: the porpoise morbillivirus (PMV), first isolated from harbor porpoises (*Phocoena phocoena*) from Northern Ireland [1], the dolphin morbillivirus (DMV), first isolated from Mediterranean striped dolphins (*Stenella coeruleoalba*) [2,3], and the pilot whale morbillivirus (PWMV), recovered from a long-finned pilot whale (*Globicephala melas*) stranded in New Jersey, USA [4] (Figure 1). Recently, three new strains were detected by reverse transcription polymerase chain reaction (RT-PCR), one in a Longman's beaked whale (*Indopacetus pacificus*) from Hawaii, one in a Guiana dolphin (*Sotalia guianensis*) from Brazil and one in two Indo-Pacific bottlenose dolphins (*Tursiops aduncus*) from Western Australia [5,6,7] (Figure 1). Over the past three decades, cetacean morbilliviruses have caused several outbreaks of lethal disease in odontocetes (toothed whales) and mysticetes (baleen whales) around the world. 

Other important pathogens in the genus *Morbillivirus* are measles virus in humans and other primates, rinderpest and peste des petits ruminants viruses in artiodactyls, canine and phocine distemper viruses in carnivores and tentatively, a paramyxovirus from domestic cats currently named feline morbillivirus [8,9,10,11]. Morbilliviruses are lymphotropic and initially replicate in lymphoid tissue before infecting epithelial cells [12,13]. All are very contagious and cause serious disease with immunosuppression in their hosts. Cetacean and pinniped morbilliviruses were first recognized in 1988 following a series of epidemics in Northwestern Europe. A symposium in Hannover, Germany, in 1994 reviewed these events and the cross-disciplinary research conducted in several countries and laboratories worldwide at that time [14,15]. Twenty years later in August 2014, a Research and Policy for Infectious Disease Dynamics (RAPIDD) workshop was convened at Princeton University, USA, to discuss the disease outbreaks and findings since then, and identify future directions for research. As a product of that workshop, here we review the antigenic, molecular, pathological and epidemiological characteristics of CeMV worldwide and discuss topics for further research. 

## 2. Antigenic and Molecular Characteristics of CeMV

**Figure 2:**
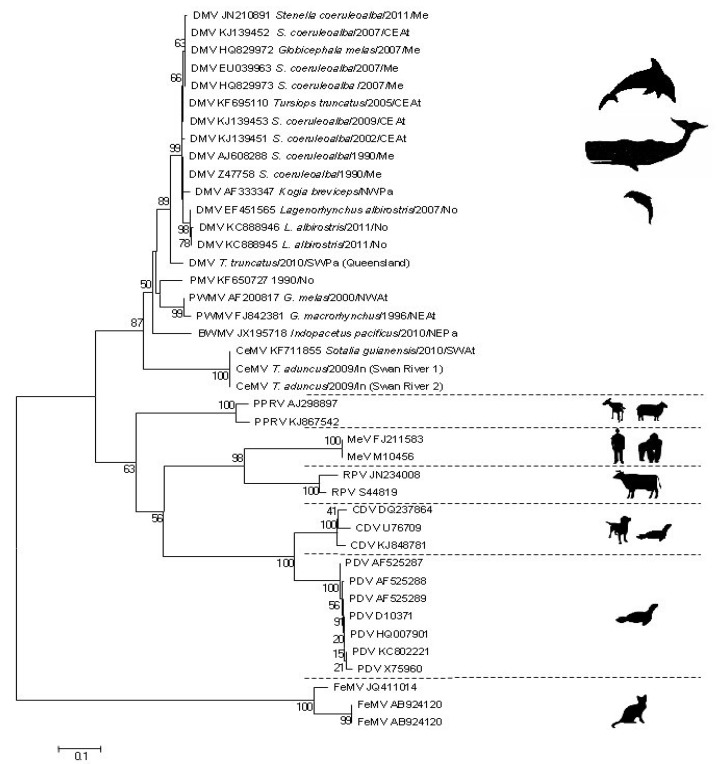
Phylogenetic analysis of a fragment of the morbillivirus P gene. Sequences were trimmed to include all sequence data available. Each sequence is denoted by its accession number (where available) and strain/isolate details (cetacean species, and year and geographic area of stranding). The evolutionary history of the isolates assessed was inferred using the neighbour-joining method with branch lengths in the same units as those of the evolutionary distances used to infer the phylogenetic tree. The evolutionary distances were computed using the Kimura 2-parameter method and are in the units of the number of base substitutions per site detailed with bootstrap values of >50 being shown against key nodes. The phylogeny includes 41 nucleotide sequences with a total of 253 positions in the final dataset. Evolutionary analyses were conducted in MEGA5 [35]. Abbreviations are: BWMV, beaked whale morbillivirus, DMV, dolphin morbillivirus; CeMV, cetacean morbillivirus; PMV, porpoise morbillivirus; PWMV, pilot whale morbillivirus; PPRV, peste-des-petits-ruminants virus; RPV; rinderpest virus; CDV, canine distemper virus; PDV, phocine distemper virus; MeV, measles virus; NEAt, Northeastern Atlantic Ocean; CEAt, Central Eastern Atlantic Ocean; NWAt, Northwest Atlantic Ocean; SWAt, Southwest Atlantic Ocean; Me, Mediterranean Sea; No, North Sea; NEPa, Northeastern Pacific Ocean; NWPa, Northwest Pacific Ocean; SWPa, Southwest Pacific Ocean; In, Indian Ocean.

Morbilliviruses are unsegmented, linear negative-sense, single-stranded RNA viruses. The DMV genome is 15,702 nucleotides long and consists of six transcription units that encode six structural proteins, the nucleocapsid protein (N), phosphoprotein (P), matrix protein (M), fusion glycoprotein (F), haemagglutinin glycoprotein (H) and the RNA-dependent RNA polymerase (L), as well as two virulence factor proteins (C and V) [9,16,17,18,19,20]. PMV and DMV are antigenically closely related, showing a similar reaction pattern with monoclonal antibodies (MoAb) raised against CDV, phocine distemper virus (PDV), peste des petits ruminants (PPRV) and rinderpest (RPV) proteins [21,22]. Serological surveys performed in cetaceans from the US and Europe showed that mean antibody titers were consistently similar to both DMV and PMV [22,23,24,25,26]. PMV and DMV are antigenically more closely related to the ruminant morbilliviruses and measles virus (MV) than to the distemper viruses [15]. Sequencing of the P, N, F and M genes further demonstrated and confirmed that PMV and DMV are closely related and that they form a separate group within the *Morbillivirus* genus, closer to the ruminant viruses and MV than to the CDV/PDV group (Figure 2) [9,16,17,18,20,27]. There is a higher (18.3%) divergence between PMV and DMV at the level of the C-terminal end of the N gene, a hypervariable domain, than between different MV isolates [17,18,28]. However, in other F and N gene regions there are fewer differences between these strains than between strains of CDV [18,20]. Thus, the present consensus is that PMV and DMV represent two strains of CeMV [16,18,20,29]. Analyses of partial P and N gene sequences of a morbillivirus (PWMV) recovered in a long-finned pilot whale (*Globicephala melas*) from New Jersey, USA, suggested that it belongs to the CeMV lineage but is distinct from PMV and DMV, and that it should be considered as a third strain of CeMV [4]. Sequence analysis of the N, P, F and H genes of another isolate from a short-finned pilot whale (*Globicephala macrorhynchus*) stranded on the Canary Islands in the Central Eastern (CE) Atlantic showed 97% homology with the *G. melas* PWMV and further confirmed a distinct strain circulating among pilot whale species [30]. However, pilot whales are also susceptible to infection by DMV [27,31]. *G. melas* and *S. coeruleoalba* that died along the coasts of Spain during the 2006–2008 Mediterranean epidemic were both infected by DMV strains that showed 100% identity across the H gene [27] and 99.9% identity over 9050 bp [31]. Similarly, P gene fragments of isolates recovered from *T. truncatus* and *G. melas* stranded along the Mediterranean coast of France in 2007–2008 were 100% identical to the Spanish *G. melas* isolate [32]. Altogether these results indicated that the same DMV strain circulated in the Mediterranean Sea and infected different cetacean species during the 2006–2008 outbreak. Furthermore, sequences of DMV strains recovered from Mediterranean cetaceans during the 2006–2008 epidemic and from *S. coeruleoalba* washed ashore in the Canary Islands in 2002–2011 were highly conserved across the short genome region characterized (Figure 2) [33]. However, there was only 99.4% and 99.3% identity between the isolates form the 1990–92 epidemic and those from the 2006–2008 events based on the nearly complete genomes (9050 bp) [19,31]. Thus, these data suggest that the 1990–1992 strain was not maintained in the Mediterranean Sea between the epidemics, and that the strain circulating in the CE Atlantic Ocean was introduced in the Mediterranean Sea in 2006. The DMV Mediterranean strains are less closely related to the isolates recovered from *L. albirostris* stranded in Germany and the Netherlands in 2007–2011 (Figure 2), suggesting that this North Sea strain did not play a role in the epidemics [31,34]. However, further research is needed to better understand the circulation of CeMV in European waters.

Very little is known about the three new CeMV related strains recently detected in odontocetes from Hawaii, Brazil, and Australia [5,6,7]. However, recent sequencing data of the P gene of the isolates recovered from two *T. aduncus* from the west coast of Australia (Indian Ocean) and from a *S. guianensis* from Brazil suggest that they differ significantly from the DMV, PMV and PWMV strains [6,7] and may represent another CeMV lineage (Figure 2). The beaked whale morbillivirus (BWMV) clusters with the ‘old’ CeMV lineage and should be considered as a new strain of this lineage (Figure 2). Sequences from a fragment of the P gene revealed that it has 86% similarity to DMV and 84% similarity to PWMV [5]. We propose to use the terminology CeMV-1 for the ‘old’ lineage that includes DMV, PMV, PWMV and BWMV and CeMV-2 for the ‘new’ lineage that includes the *T. aduncus* and *S. guianensis* morbilliviruses until the taxonomy of these viruses is further explored. 

The close genetic relationship between cetacean and ruminant morbilliviruses has led to the suggestion that they may have a common ancestor [7,16]. Closely related to the hippopotamus (*Hippopotamus amphibius*), cetaceans belong to the clade Cetartiodactyla [36,37]. As several species of this clade are susceptible to RPV and PPRV [38,39], it is possible that a host jump occurred between a cetacean and another member of the Cetartiodactyla, and that ecological isolation led to distinct virus species. The presence of similar host proteins and cell receptors in cetaceans and artiodactyls may favour cross-species transmission [9,13,40]. However, further studies are needed to confirm this hypothesis.

## 3. Mechanisms of Cellular Entry and Receptors

The H glycoprotein is responsible for virus attachment to the host cell membrane and for cellular entry. The F glycoprotein causes fusion with the host cell membrane and, together with the M protein, invokes cell-to-cell fusion [20,41]. H and F interact with cellular receptors that allow virus entry and determine host susceptibility, tissue tropism and viral pathogenesis [12,42]. The signaling lymphocyte activation molecule (SLAM or CD150) and the poliovirus like receptor 4 (PVLR4 or nectin 4) have both been recently identified as the major receptors for wild-type morbilliviruses in immune and polarized epithelial cells, respectively [9,13,42,43,44,45]. Besides, CD147, a transmembrane glycoprotein that belongs to the immunoglobulin family and is present on a variety of cells including neuronal and endothelial cells, and the membrane bound form of heparin binding epithelial growth factor have been suggested to function as entry receptors for MeV and PDV, respectively [42,46]. Most morbilliviruses, including MV, CDV, PDV, PPRV, and RPV use the SLAM of their respective host species as a receptor [42,43,47,48]. 

Phylogenetic trees based on the structure of the SLAM and H proteins indicated that they co-evolved [9]. The SLAM receptors have immunoglobulin-like variable (V) and constant-2 (C2) domains in their extracellular regions with the V domain providing an interface for the morbillivirus H glycoprotein [49]. Substitution in the amino acid residues of this interface may lead to a loss of, a reduction in, or an increase in, viral infectivity [13]. The morbillivirus H glycoprotein displays a strong affinity for this domain in its respective host [13]. However, a recent study showed that only one amino acid exchange in H was required for functional adaptation of CDV to the human SLAM cell receptor *in vitro* [50]. The SLAM receptor has been characterized in seven species of mysticetes and in 19 species of odontocetes [9,13]. Three-dimensional homology models showed that there are 32 amino acid residues on the interface of cetacean SLAM that may contribute to morbillivirus binding [13]. The similarity of the 32 residues was higher between the cetacean and cow SLAMs (26 amino acid residues) than between the Pacific white-sided dolphin (*Lagenorhynchus obliquidens*) and the spotted seal (*Phoca largha*) SLAM (21 amino acid residues) [9,13], as would be expected based on the host relationships. Among the nine cetacean families examined, variations were found between six amino acid residues, with charge alterations for four of them [13]. Interestingly, three residue substitutions (G68, H90 and H130) that introduced charge alteration and possible change in viral affinity were observed in the SLAM of the Delphinidae, while these residues were mostly conserved in the receptor of the other cetacean families [13]. As morbillivirus mass mortalities have mostly been detected in the Delphinidae, it is possible that their SLAMs have a higher affinity for CeMV resulting in increased viral infectivity and dissemination [13]. Among the Delphinidae, only *T. truncatus*, *T. aduncus* and, *S. coeruleoalba* had variation at position 130 [13] and during CeMV outbreaks mass die-offs were overwhelmingly dominated by these species [7,51,52]. The only other odontocete that presented this H130Q variation was *P. phocoena*, a species that was affected by morbillivirus infection in 1988–1990 [53,54]. Further studies are needed to confirm if the SLAM of dolphins, porpoises and whales is indeed the immune cell receptor for CeMV and should investigate whether alternate potential receptors, such as nectin4 and CD147, are present on the cells of these mammals.

## 4. Diagnosis

Though virus isolation remains the gold standard for definitive diagnosis, it is challenging when dealing with stranded cetacean carcasses. RT-PCR followed by sequencing has proven very helpful for obtaining rapid confirmation of CeMV infection, to differentiate between PMV and DMV and to identify new strains [4,5,6,7,16,20,55]. Histology and immunohistochemistry have provided further confirmation of the disease and insights into its pathogenesis and have permitted differentiation between systemic disease and localized chronic infection of the central nervous system (CNS) [56,57,58,59,60]. Serological studies have also been useful for studying CeMV epidemiology, to assess the immune status of populations before and after an outbreak and to predict the occurrence of new epidemics [21,23,24,26,61,62,63,64,65]. 

### 4.1. Histology and Immunohistochemistry

Classical histological techniques have been used to investigate CeMV disease and pathogenesis since the first *P. phocoena* and Mediterranean *S. coeruleoalba* were suspected of dying of morbillivirus infection [2,53]. Immunohistochemistry (IHC) has greatly enhanced the sensitivity and specificity of histopathological diagnosis by enabling the detection of morbillivirus antigen in cases where tissue preservation is poor or where classical lesions have been obscured by opportunistic pathogens. IHC studies have been conducted by using a commercially available MoAb for CDV N protein [5,6,7,33,66,67,68,69], a MoAb for PDV hemagglutinin [2,53,57], or a rabbit polyclonal antiserum to rinderpest virus [70]. Together with RT-PCR, these techniques recently permitted the identification of morbillivirus outbreaks in *T. truncatus* and *T. aduncus* from South Australia in 2013 [51], and in *T. truncatus* from the NW Atlantic, ongoing since 2013 [71]. At the time of writing specific MoAb for DMV or PMV proteins are not commercially available although they would be useful for accurate diagnosis and research in the future. Histology and IHC techniques should always be used to confirm the molecular diagnosis of systemic morbillivirus infection during an outbreak of mortality.

### 4.2. Virus Isolation

The isolation of DMV and PMV has been achieved using homogenates of lung tissue from *S. coeruleoalba* and *P. phocoena* inoculated onto monolayers of African green monkey kidney (Vero) cells following standard methodologies [1,3,22]. Primary canine kidney epithelial cell cultures, bovine foetal lung cells and *T. truncatus* peripheral blood mononuclear cells have also proved useful for isolation of CeMV directly or after co-cultivation with Vero cells [1,17,21]. Primary culture of kidney cells derived from diseased *P. phocoena* permitted direct virus isolation [22]. Repeated passages of the inoculated cell cultures and, consequently, several weeks are typically needed before virus growth can be detected [17,22]. Recently, Vero cells expressing the canine SLAM (Vero.DogSLAMtag cells) were shown to reduce the time necessary for PDV isolation from weeks to days [72]. These cells were also successfully used to grow stocks of PMV and DMV initially passaged on Vero cells and to isolate DMV from the brain of a *G. melas* stranded in Valencia during the 2006–2008 epidemic [27,55,67]. More recently, they proved useful to isolate CeMV from fresh tissues as part of the investigation into the *T. truncatus* morbillivirus outbreak along the eastern Atlantic coast of USA in 2013 [71]. Virus isolation has the added benefit of providing antigen necessary to carry out serological testing, as described in the serology section below. It may also provide genomic material for more complete phylogenetic analysis.

### 4.3. Serology

Virus neutralization (VN) tests, plaque reduction (PR) assays and indirect enzyme-linked immunosorbent assays (iELISAs) are the main platforms used to detect antibodies against CeMV. The iELISA allows the detection of antibodies directed against the N, P, F and H CeMV proteins [73] whereas only antibodies to the surface glycoproteins (H and F) are detected by the VN and PR assays [16]. Morbilliviruses are antigenically closely related and may cross-neutralize one another. However, serum raised against one morbillivirus will neutralize the homologous virus at a higher titer than it will heterologous morbilliviruses [63,74]. Thus, when working with cetaceans it is very important to use CeMV strains in the serological tests to avoid false negatives. 

Indirect ELISAs were developed to analyze hemolyzed serum samples that could be cytotoxic and, as such, could prevent the detection of morbillivirus antibodies at low dilutions in virus neutralization tests [61,62]. These assays used whole DMV [61,62,63] or the recombinant N protein of RPV [75] to detect morbillivirus antibodies. Cetacean antibodies were detected using horseradish-peroxidase-conjugated protein A, a cell wall constituent of *Staphylococcus aureus* that binds non-specifically the immunoglobulins of several species of vertebrates including odontocetes [21,76]. Recently, purified DMV-N protein expressed from a baculovirus (*Autographa californica nuclear polyhedrosis virus*) vector was used as the coating antigen in the iELISA and permitted the detection of morbillivirus antibodies in the sera of odontocetes [77]. The iELISA appears to be more sensitive than the classical VN test and may be useful as a serological tool for the mass screening of morbillivirus antibodies in cetaceans. A competitive ELISA using MoAbs to CDV and PDV was developed for testing sera from various species of marine mammals. Its main advantage over iELISAs is that a single anti-mouse immunoglobulin conjugate can be used on serum from any animal species [74]. However, sensitivity was lower for detection of cetacean compared to carnivore morbilliviruses [74].

The VN test is highly sensitive and very specific and is considered the most reliable assay for the detection of CeMV antibodies [74]. Antibody titers are expressed as the reciprocal of the highest dilution of sera that completely neutralizes cytopathic effects. Titers of 1:16 or higher are considered to be indicative of exposure to CeMV, although higher thresholds can be used to reduce the likelihood of false positives. A more conservative interpretation is recommended when either new host species or new geographic areas are under investigation. A PR assay was developed to allow detection of antibodies in hemolyzed sera [78,79]. In this test, titers are expressed as the reciprocal of the highest dilution that gave 80% reduction in the number of plaques compared to the negative control [79]. Although Vero cells are most commonly utilized in these tests, use of Vero. DogSLAMtag cells, which allows for improved virus replication and permits reduction of incubation time from nine days to four days ([72]; Saliki, unpublished observations) may be a more robust and cost-effective alternative. 

### 4.4. Reverse Transcription Polymerase Chain Reaction

A “universal” morbillivirus primer set, based on highly conserved regions of the morbillivirus P gene identified by Barrett *et al*. [16] has been successfully used to detect CeMV by RT-PCR during outbreaks worldwide [6,7,68,70,75]. Using a similar approach, Krafft *et al*. [80] designed a protocol that allows amplification of degraded RNA in formalin-fixed paraffin embedded samples and in unfixed autolyzed tissues. This technique was useful to confirm CeMV infection in fixed tissues from *D. delphis* stranded along the coast of California in 1995–1997 and in cases of chronic encephalitis in Mediterranean *S. coeruleoalaba* [56,75]. Since then, other primers including sets of “universal” morbillivirus primers based on the conserved N terminus of the morbillivirus N gene, were also successfully used to detect CeMV [67]. A real-time RT-PCR (rtRT-PCR) that targets the hypervariable C terminal domain of the N gene was developed by Grant *et al*. [55] for a rapid and differential detection of dolphin and porpoise morbilliviruses. This test is rapid, very sensitive and specific for either DMV or PMV and does not cross-react with CDV, PDV, RPV, PPRV and MV [55]. A rtRT-PCR assay that targeted the glyceraldehyde 3-phosphate dehydrogenase (GAPDH) gene, as a house-keeping gene, was developed to determine whether total RNA extracted from stranded cetacean tissues is amplifiable [55]. This test allowed for the detection of GAPDH gene sequences from 14 marine mammal species and is essential for interpreting negative results with the morbillivirus RT-PCRs. Another rtRT-PCR was later designed to amplify a highly conserved region within the F gene and to differentiate between DMV, PMV, and PWMV [81,82]. More recently, a pan-marine mammal morbillivirus semi-nested RT-PCR using a degenerate set of primers targeting conserved sequences of the P gene was described [33] for the detection of both pinniped and cetacean morbilliviruses. Such an assay is useful for detecting morbilliviruses in multiple marine mammal species. The L primers, described by Woo *et al.* [11], may also be helpful for detecting CeMV in odontocetes and mysticetes. Clearly, with all the advances in molecular biology, diagnosing CeMV infection has become much faster, easier and more reliable. RT-PCR assays should be used together with the other techniques to distinguish among acute infection, prolonged persistence of morbillivirus RNA following CeMV acute disease, and chronic infection. When CeMV infection is detected in a novel host species, samples should be sequenced for species confirmation and identification and also sent to morbillivirus reference centers for genetic confirmation of the species involved. 

## 5. Pathology and Pathogenesis of CeMV Infection

Most morbilliviruses are lymphotropic and epitheliotropic [12]. After initial replication in the lymphoid tissues, the virus is disseminated by infected lymphocytes through the lymphatic system and spreads to epithelial cells [12,83,84,85,86]. Histology and immunohistochemistry data indicate that CeMV-associated pathology resembles that commonly seen in other morbillivirus infections in animal and human hosts [54]. 

**Figure 3:**
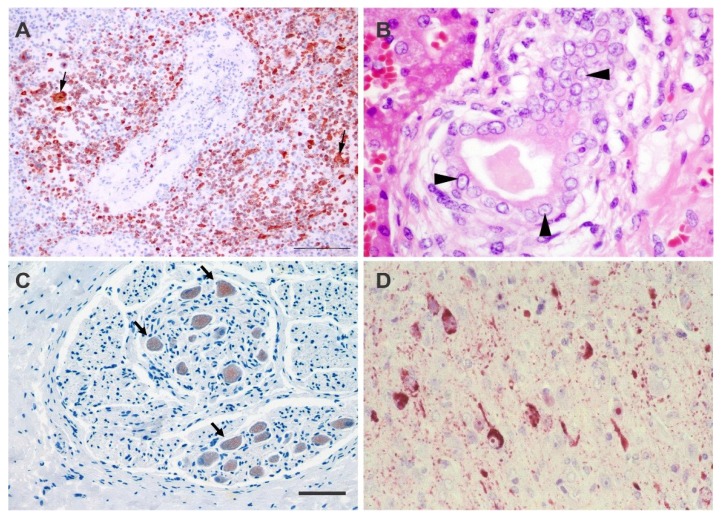
(**a**) Bottlenose dolphin (*Tursiops truncatus*), pulmonary lymph node, DMV infection, Canary Islands, Spain, 2005 (© IUSA, ULPGC). Positive intranuclear and intracytoplasmic immunoperoxidase staining of morbilliviral antigen in mononuclear and multinucleated giant syncytial cells (arrows). Avidin-biotin-peroxidase with Harri’s hematoxylin counterstain × 250 (20 × objective); (**b**) Common bottlenose dolphin (*Tursiops truncatus*), liver and bile ductule, DMV infection, United States, 2014 (© K. Colegrove). Small eosinophilic intranuclear inclusions within biliary epithelial cells (arrows). ×750 (60 × objective); (**c**) Guiana dolphin (*Sotalia guianensis*)*,* glandular stomach, CeMV infection, São Mateus, Brazil, 2010. Granular immunohistochemical staining of morbilliviral antigen in neuronal cytoplasm in the myenteric plexus. Avidin-biotin complex immunoperoxidase technique, Mayer’s haematoxylin counterstain. Bar = 50 µm. (© Katia Groch); (**d**) Striped dolphin (*Stenella coeruleoalba*), brain, DMV infection, Latium, Italy, 1993 (© G. Di Guardo). Strong immunohistochemical labeling of morbilliviral antigen in cortical neurons, intranuclear viral inclusion bodies and in the surrounding neuropil. Avidin-biotin peroxidase technique with Mayer’s haematoxylin counterstain ×500 (40 × objective).

### 5.1. Acute, Systemic Disease

Acutely fatal CeMV infection is generally associated with severe multifocal to diffuse interstitial broncho-pneumonia characterized by necrosis of type I pneumocytes and bronchiolar epithelial cells, interstitial oedema, type II pneumocyte hyperplasia, and formation of large syncytia in the alveolar and bronchiolar lumina. Intracytoplasmic and intranuclear inclusion bodies can be noted and are sometimes numerous in respiratory epithelia, bronchiolar gland epithelia and the syncytial cells. Generalized lymphoid depletion with germinal center necrosis is usually present and syncytial cells (Warthin-Finkeldey type) are often prominent in lymphoid tissues (Figure 3A). There may be evidence of viral replication (inclusion bodies) in epithelia and neural cells of other body systems (Figure 3B,C). Multifocal non-suppurative encephalitis may also be present (Figure 3D). Therefore, there may be strong IHC staining in the lungs, lymphoid organs (Figure 3A) and other tissues that is variable in extent between individual cases [7,54,57,59,60,68,87]. 

### 5.2. Sub-Acute Systemic Disease

Animals that survive the acute stage of infection may succumb to opportunistic infections (*Toxoplama gondii*, herpesviruses, bacteria such as *Photobacterium damselae*, and fungi) as a consequence of the profound immunosuppression. This typical pattern has been commonly seen in odontocetes that died during outbreaks of CeMV in Europe, South America, the USA and Australia [6,7,32,57,58,59,60,68,88,89,90,91]. While some of the lesions typical of acute infection may no longer be present or be largely obscured by the inflammatory response to the opportunistic pathogens, non-suppurative demyelinating meningoencephalitis (Figure 3D), often focally distributed, is a feature of sub-acute infection. Colonization of the brain by opportunistic mycotic pathogens (e.g., *Aspergillus* spp.) is also common [7,59]. IHC and RT-PCR are useful for confirmation of the diagnosis of morbilliviral infection in these cases.

### 5.3. Chronic Systemic Infection

Animals may survive the acute and sub-acute manifestations of infection but succumb sometime later to the secondary infections acquired as a result of viral immunosuppression, or from complications of CNS infection. Typically these animals are in poor body condition at the time of death and the proximate cause of death may be multifactorial. Invariably there are no or few lesions directly attributable to CeMV but viral antigen may be detectable by IHC in some lymph nodes and lungs [57] and viral RNA may be amplified by RT-PCR [57,92]. 

This chronic form of infection was a feature of the 1987–1988 *T. truncatus* epizootic off the US Atlantic coast, where confirmation of morbillivirus infection was only possible using IHC (53% of 79 cases) [57] and RT-PCR (86% of 29 IHC negative cases) [92]). More recently, chronic CeMV infection was detected in two *T. aduncus* from Western Australia [7]. It was characterized by pronounced lymphoid depletion and severe secondary infections and the almost complete absence of typical morbillivirus lesions in the lungs and brain. Morbillivirus antigen was detected in lymphoid tissues, as well as in the hepatic sinusoidal endothelial cells and Kupffer cells, biliary epithelium, and tunica media myocytes of blood vessels within the liver and mesenteric lymph nodes but not in the lungs or brain [7]. RT-PCR amplified morbilliviral RNA in the same tissues. These results suggested that the dolphins had survived the acute phase of the infection but died following profound immunosuppression and secondary infections [7]. If the pathogenesis of CeMV is similar to that of MV [93], cetaceans that survived acute and sub-acute infection could show prolonged RNA persistence in the blood and lymphoid organs and could be RT-PCR positive in the absence of typical morbillivirus lesions. The concurrent use of histology, IHC and molecular techniques is recommended to further explore the pathogenesis of chronic systemic infections.

### 5.4. Chronic, Localized CeMV Encephalitis 

Cetaceans that have cleared and resolved DMV systemic infection may develop a CNS form that is characterized by the presence of lesions and virus only in the brain [33,56,66,94]. This CNS form was consistently observed in *S. coeruleoalba* after the two epidemics in the Mediterranean Sea [56,94]. By contrast with the sub-acute cerebral CeMV infection, cytoplasmic or nuclear eosinophilic inclusions were only occasionally detected and syncytial cells were not observed in the CNS form. Many neuronal processes showed immunostaining for CeMV, and some areas had massive accumulation of CeMV-antigen, while contiguous zones of the brain had almost no staining. This suggests that the presence of CeMV was more the result of cell-to-cell spreading of infection rather than of a multifocal infection indicative of blood-borne infection. The CNS form appears to share histological characteristics with subacute sclerosing panencephalitis (SSPE) and old dog encephalitis (ODE), chronic latent localized infections that affect humans and dogs, respectively, and are caused by defective forms of MV and CDV, respectively [95,96,97]. As in SSPE and ODE, the CNS lesions were localized predominantly in the cerebral cortex, subcortical white matter, and the thalamus, while the cerebellum was mostly spared. In the three conditions perivascular cuffing, diffuse gliosis, and glial nodules with neurophagia were the most prominent changes [94]. Focal malacia was not detected [94,96,98,99,100]. Demyelination was less prominent in dolphins with the exclusively CNS presentation and in dogs with ODE than is seen in the meningoencephalitis of CeMV or CDV, respectively [56,66,94,101,102]. As in the human and canine presentations, antigen and viral RNA could be detected in dolphin brains but the virus proved difficult to isolate [101]. The mechanism for this is unknown but delayed clearance of antigen and RNA from the CNS may be related to reduced immune surveillance in an immuno-privileged site [99,100]. RT-PCR studies on the brain of *S. coeruleoalba* chronically affected by CeMV suggest that the sequence of the P gene is different in these cases [56], but further research is needed. The role of CD147 and other cell receptors in the pathogenesis of this form of the disease should be further examined [103].

The prevalence of the DMV-CNS form in *S. coeruleoalba* seems to be higher in dolphins than prevalence of SSPE in humans. Indeed, in a hypothetical scenario where the whole central Mediterranean *S. coeruleoalba* population (15,778 individuals; 95% CI = 10,940 to 22,756 [104]) was exposed to DMV during the 2006–2008 epidemic, the proportional morbidity rate of CNS-localized infection would be between 141 and 293 per 100,000 cases [56]. In a recent study in Germany, the risk of developing SSPE after acute measles infection in children below five years of age was estimated to range from 1:1700 to 1:3300 (30.3 to 58.8 per 100,000 cases) in 2003–2009 [105]. Further research should explore true age-weighted prevalence of this syndrome in dolphins. There are no comparable data for ODE though it is assumed to be a very rare complication of CDV infection. The significance of this related to epidemiology remains to be determined. However, the diversity of cetacean morbilliviruses seems greater than that seen within other morbillivirus species. This is unexpected for a virus that induces lifelong immunity and high cross-protection between congeneric viruses in long-lived hosts. It is possible that CNS persistence plays a role in the maintenance of strains in an ocean basin, although, as a dead end infection, it is unlikely to contribute to virus transmission to other cetaceans.

Although the CNS form has been mostly described in Mediterranean *S. coeruleoalba*, a similar presentation was described in a few other sporadic cases, namely a mature *L. obliquidens* stranded on the coast of Miyasaki, Japan, in March 1998 [106], in a juvenile white-beaked dolphin (*Lagenorhynchus albirostris*) that beached on the island of Ameland, the Netherlands, in June 2011 and died in a rehabilitation center six months later [34], in four *S. coeruleoalba* stranded along the coasts of the Canary Islands in 2002–2011 [33], and in several *T. truncatus* from North America (Colegrove, pers. observation). The CNS form was not detected in *P. phocoena* from the North Sea and Northeastern (NE) Atlantic, although the brains from relatively few animals were examined [107]. Current data would suggest that of the three known strains of CeMV, the DMV variant is the only one associated with the chronic CNS presentation. However, it is also the most prevalent variant detected in nature, and much more research would be required before conclusions could be drawn.

### 5.5. Subclinical Infection

The pathogenesis and clinical course of morbillivirus infections in cetaceans are poorly understood as there are no comparable laboratory studies to those on CDV in dogs and MV in primates [85,108]. Acute and subacute systemic presentations and chronic CNS infection causing death have been documented, as described in preceding sections. However, the existence and nature of subclinical infection remains speculative. Between 1995–1997, a series of DMV seropositive (> 1:50) *D. delphis* beached along the southern California coast [75]. One of the six dolphins survived and developed very high titers (1:720) against DMV while in rehabilitation. The other five were euthanized and, at necropsy, none had classical morbillivirus lesions. However, one had a mild lymphocytic meningoencephalitis and its brain was positive for morbillivirus RNA by RT-PCR, as described in Mediterranean *S. coeruleoalba*. Morbilliviral RNA was also detected in the spleen and heart of two other dolphins without histological lesions [75]. Whether this actually represents subclinical infection in *D. delphis* or an atypical viral strain/host presentation in the eastern Pacific is unknown.

Similarly, a low prevalence of serum antibodies in apparently healthy live-captured *T. truncatus* from the Indian River Lagoon, on Florida’s Atlantic coast, without a prior increase in mortalities in the population was suggestive of virus circulation in the absence of an epidemic and thus, of subclinical infection [64]. However, this is a complex system with documented evidence of repeated CeMV epidemics over a period of at least 1982 to 2013 [25,57,72]. Thus, a better understanding of CeMV pathogenesis and immunity in dolphins is required before we can adequately interpret findings from field investigations.

### 5.6. Immune Function and CeMV Infections

Morbillivirus infections have long been known to result in host immune suppression [109,110,111,112]. Recent studies suggest similar effects in naturally infected, wild *T. truncatus* sampled as part of capture-release studies for health assessment. Bossart *et al*. [65] reported a significant decrease in mitogen-induced T cell proliferation along with an increase in lysozyme concentrations and a marginally significant increase in monocyte phagocytosis, along with a marginally significant decrease in the numbers of CD4+ T lymphocytes in *T. truncatus* that had antibody titers ≥ 1:8 against morbillivirus, suggestive of previous exposure to the virus, compared to animals with lower or no titers. They found no effects on neutrophil phagocytosis. While the timing of the morbillivirus infection (active infection, chronic infection, resolved past infection) cannot be determined from titers alone, it is clear that there is an association between modulation of immune functions and previous exposure to morbillivirus infection in *T. truncatus*, as observed in other species. Further studies are needed.

### 5.7. CeMV Transmission

#### 5.7.1. Horizontal Transmission

Morbillivirus transmission is thought to occur mostly after the inhalation of aerosolized virus shed by infected individuals [113]. This horizontal transmission is likely to occur among cetaceans too and to be favored by a gregarious behavior and a high density of cetaceans [67,114]. Transmission by inhalation of expired blowhole droplets possibly occurs during breathing in a synchronized fashion when large numbers of tightly grouped cetaceans are travelling and feeding together or are engaged in social activities [20,115,116].

#### 5.7.2. Evidence for Vertical Transmission

Morbillivirus antigen was detected in the mammary gland of *T. truncatus* from the US Atlantic coast epidemic in 1987–1988 and of *S. coeuruleoalba* from the Mediterranean Sea outbreak in 1990 [59,87] and in the penile and preputial epithelium of a *P. phocoena* from the North Sea [107]. Furthermore, a testicular fibroma collected in a short-beaked common dolphin (*Delphinus delphis* *ponticus*) from the Black Sea was positive for morbillivirus RNA by RT-PCR [117]. The first evidence that vertical transmission may occur was the detection of morbilliviral RNA in the brain, lung, spleen, lymph node, and liver from the seven-month fetus of a DMV-infected *G. melas* stranded in the Balearic Islands in 2007 [88]. These data suggest that CeMV infected females may transmit the infection to their fetuses and neonates *in utero* and during lactation, respectively. *In utero* transmission has been reported for MV in humans. The effects on the fetus depend on the stage of pregnancy and include abortion, *in utero* death or premature birth [118]. When MV infects pregnant women in the peri-natal period, neonates are at risk of congenital measles and have a higher risk of developing early and fulminant SSPE because of the incomplete transfer of protective antibodies [96,119]. A similar situation may have happened in a *S. coeruleoalba* calf stranded in Italy in November 2009 with a CNS infection [120] and in a neonate sperm whale (*Physeter macrocephalus*) washed ashore in Oahu, Hawaii in May of 20l1 [121]. 

## 6. Outbreaks of Disease and Epidemiology

Morbilliviruses are extremely infectious and are likely to infect most of the immunologically naive individuals in a population. Herd formation and migration increase the probability of transmission [37,113]. Morbilliviruses require large populations of susceptible individuals (e.g., 300,000 for measles virus in humans) to persist endemically, as there is no carrier state and infection confers lifelong immunity [113]. However, the persistence of morbilliviruses in relatively small (possibly multispecies) host metapopulations remains an important unsolved problem in disease ecology [122]. Newborn individuals typically have maternal immunity if their mothers had previously been infected. After some months, this immunity is lost and the young individuals are fully susceptible to infection [123,124]. 

CeMV infection has been detected using various techniques in several species of odontocetes and mysticetes worldwide (Table 1). DMV is the strain most commonly observed in cetaceans from the Northern Hemisphere, followed by PMV and PWMV (Table 1). Serological studies strongly suggest that CeMV is endemic in gregarious odontocete species in the North Atlantic and, possibly in the Southwestern Atlantic and in the South Pacific [23,24,25,61,63]. Pilot whale (*Globicephala* spp.), dusky dolphin (*Lagenorhynchus obscurus*), Fraser’s dolphin (*Lagenodelphis hosei*) and melon-headed whale (*Peponocephala electra*) populations had high prevalences of DMV-seropositives and may be reservoirs and vectors of the infection to susceptible species [23,24,25,62,63,90]. In the absence of, or decrease in, herd immunity, outbreaks of lethal disease may occur in susceptible species, as has repeatedly been observed in Europe, the Americas, and Australia since the late 1980s. 

**Table 1:** CeMV infection in odontocetes and mysticetes worldwide. Abbreviations are: VI = virus isolation, IHC = immunohistochemistry, S = serology, RT-PCR = reverse-transcriptase polymerase chain reaction, PMV = porpoise morbillivirus, CeMV = cetacean morbillivirus, DMV = dolphin morbillivirus, PWMV = pilot whale morbillivirus and CeMV, NL = new lineage of CeMV.

Ocean Provinces/Species	Years	Countries	Epidemiolo-gical Status	Diagnosis	Virus	Literature Cited
***Eastern Atlantic & North Sea***						
*Phocoena phocoena*	1988–1990	N. Ireland, UK, Netherlands	periodic mortalities	VI, IHC, S, RT-PCR	PMV	[1,22,53], [54,61,63]
*Delphinus delphis*	1988–1990	UK, Netherlands	unknown	S	CeMV	[22,61,63]
*Lagenorhynchus albirostris*	1988–1990, 2007, 2011	Germany, Netherlands	periodic mortalities	S, IHC, RT-PCR	DMV	[22,34,61,125]
*Balaenoptera physalus*	1983	Iceland	unknown	S	CeMV	[17]
*B. physalus*	1997–1998	Belgium, France	periodic mortalities	IHC	unknown	[126]
*Tursiops truncatus*	1999	Kent, UK	unknown	S	CeMV	[63]
*Globicephala macrorhynchus*	1996	Canary Islands	periodic mortalities	RT-PCR	PWM	[30]
*T. truncatus*	2005	Canary Islands	periodic mortalities	IHC, RT-PCR	DMV	[69]
*S. coeruleoalba*	2002–2011	Canary Islands	periodic mortalities	IHQ, RT-PCR	DMV	[33]
*D. delphis*	2007	Canary Islands	periodic mortalities	IHQ, RT-PCR	DMV	[33]
***Mediterranean Sea***						
*S. coeruleoalba*	1990–1992	Spain, France, Italy, Greece	epidemic	VI, IHC, S, RT-PCR	DMV	[2,3,21,58,127]
*S. coeruleoalba*	2006–2008	Spain, France, Italy	epidemic	IHC, RT-PCR	DMV	[32,66,67]
*T. truncatus*	1994; 2007–2008, 2011	Israel, Spain, France, Italy	periodic mortalities	IHC, RT-PCR, S	DMV	[32,63,66,128]
*D. delphis*	1990	Italy	unknown	S	CeMV	[21]
*Globicephala melas*	2006–2007	Spain, France	epidemic	IHQ, RT-PCR	DMV	[88]
*Grampus griseus*	1997, 1999	Valencia, Spain	unknown	S	CeMV	[63]
*Balaenoptera acutorostrata*	1993	Tuscany, Italy	unknown	S	unknown	[58]
*B. physalus*	2011	Tuscany, Italy	periodic mortalities	RT-PCR	DMV	[89]
***Northwestern Atlantic***						
*T. truncatus*	1982	Florida, USA	epidemic	S, IHC	CeMV	[25,129]
*T. truncatus*	1987–1988	East coast USA	epidemic	IHC, RT-PCR	CeMV	[57,92]
*T. truncatus*	1993–1994	Gulf of Mexico, USA	epidemic	IHC, RT-PCR	CeMV	[25,57,92]
*T. truncatus*	2003–2007	Florida, USA	unknown	S, IHC	CeMV	[64,65]
*T. truncatus*	2013–2014	East coast USA	epidemic	IHC, RT-PCR	DMV	[71,130]
*T. truncatus*	1992–1994	East coast USA	endemic	S	CeMV	[25]
*G. melas*	1982–1993	Northeast coast USA	endemic	S	CeMV	[23]
*G. macrorhynchus*	1986–1994	Florida, USA	endemic	S	CeMV	[23]
*G. melas*	late nineties	New Jersey, USA	periodic mortalities	IHC, RT-PCR	PWM	[4]
*S. coeruleoalba*	1991–1993	Northeast coast USA	unknown	S	CeMV	[24]
*Stenella frontalis*	1993	Northeast coast USA	unknown	S	CeMV	[24]
*D. delphis*	1980–1994	Northeast coast USA	possibly endemic	S	CeMV	[24]
*Lagenorhynchus acutus*	1985–1993	Northeast coast USA	unknown	S	CeMV	[24]
*Kogia breviceps*	1983–1991	Southeast coast USA	unknown	S	CeMV	[24]
*Feresa attenuata*	1983	Southeast coast USA	unknown	S	CeMV	[24]
*Pseudorca crassidens*	1982–1988	Southeast coast USA	possibly endemic	S	CeMV	[24]
*Lagenodelphis hosei*	1994	Gulf of Mexico, USA	possibly endemic	S	CeMV	[24]
*P. phocoena*	1993–1994	East coast, Canada	unknown	S	CeMV	[24]
***Southwestern Atlantic***						
*L. hosei*	1999	Puerto Madryn, Argentina	unknown	S	CeMV	[63]
*L. hosei*	1999	Rio de Janeiro, Brazil	unknown	S	CeMV	[63]
*Sotalia guianensis*	2010	Espirito Santo, Brazil	unknown	IHC, RT-PCR	CeMV *NL*	[6]
***Eastern Pacific***						
*Lagenorhynchus obscurus*	1993–1995	Central Peru	endemic	S	CeMV	[62]*’*
*T. truncatus*	1993–1995	Central Peru	endemic	S	CeMV	[62]
*Delphinus capensis*	1993–1995	Central Peru	endemic	S	CeMV	[62]
*D. delphis*	1995–1997	California, USA	unknown	S, IHC, RT-PCR	CeMV	[4,75]
*Indopacetus pacificus*	2010	Hawaii, USA	unknown	HC, RT-PCR	BWMV	[5]
*Physeter macrocephalus*	2011	Hawaii, USA	unknown	RT-PCR	BWMV	[121]
***Western Pacific***							
*Lagenorhynchus obliquidens*	1998	Miyazaki, Japan	unknown	IHC	unknown	[106]
*K. breviceps*	2009	SW Taiwan	periodic mortalities	IHC, RT-PCR	DMV	[70]
*G. melas*	1997	Northland, New Zealand	endemic	S	CeMV	[63]
*T. truncatus*	1997	Tasmania, Australia	unknown	S	CeMV	[63]
*Peponocephala electra*	2005–2007	NE Australia	endemic	S	CeMV	[90]
*Tursiops aduncus*	2005–2010	NE Australia	unknown	S	CeMV	[90]
*L. hosei*	2006	NE Australia	unknown	S	CeMV	[90]
*T. truncatus*	2009–2010	Queensland, Australia	periodic mortalities	S, IHC, RT-PCR	DMV	[68,90]
***Indian Ocean***						
*D. delphis*	1999	East London, South Africa	unknown	S	CeMV	[63]
*T. aduncus*	2009	Western Australia	periodic mortalitie *s*	IHC, RT-PCR	CeMV *NL*	[7]
***Southern Ocean***						
*T. aduncus*	2012–2013	South Australia	unknown	IHC, RT-PCR	CeMV *NL*	[51,131]
*T. truncatus*	2013	South Australia	unknown	IHC, RT-PCR	CeMV *NL*	[51,131]
*D. delphis*	2012–2013	South Australia	unknown	RT-PCR	CeMV* NL*	[51,131]

### 6.1. Europe

#### 6.1.1. North Sea, NE Atlantic, and CE Atlantic

In the North Sea and NE Atlantic Ocean, the first cetacean morbillivirus mortalities were detected in *P. phocoena* stranded along the coasts of Ireland, England and the Netherlands in 1988–1990 [22,53]. Sporadic morbillivirus infections were further observed in *L. albirostris* and fin whales (*Balaenoptera physalus*) beached in Northern Europe in 1990–2011 [34,126]. Serological surveys of stranded *D. delphis* and *P. phocoena* in the UK and the Netherlands in 1988–1999 showed that prevalence of DMV-seropositivity was declining over time and that only adult porpoises and dolphins were positive in 1997–1999. This suggested that the virus had not persisted as an endemic infection in these populations [61,63]. Similarly, with the exception of a *P. phocoena* with systemic morbillivirus infection beached in Kent, UK, in late 1990, systemic morbilliviral disease was not detected in any porpoise that stranded along the coasts of Belgium, northern France, England and Germany in 1990–2000 [132,133,134,135]. Though the number of *P. phocoena* in the North Sea and adjacent waters was theoretically large enough to sustain an endemic infection (341,366 individual [95% confidence interval = 260,000–449,000] in 1994 [136]), their solitary behavior likely did not favor morbillivirus transmission and maintenance in this population. The presence of high titers of DMV antibodies in the serum of a juvenile *G. melas* collected in the English Channel in 1996 suggested that this species could be involved in the maintenance of the virus in the NE Atlantic [61]. However, further serological surveys and molecular investigations are needed to understand the ecology of CeMV in this ocean basin.

Recently, CeMV infection was detected in Delphinidae from the CE Atlantic Ocean. A virus closely related to the PWMV strain was detected by RT-PCR in the brain of a *G. macrorhynchus* stranded in Tenerife, Canary Islands, Spain, in 1996 [30]. In addition, an IHC and RT-PCR retrospective survey showed that DMV caused chronic CNS disease in *S. coeruleoalba* and *D. delphis* washed ashore in the Canary Islands in the period 2002-2011 [33]. Finally, a systemic DMV infection was observed in a *T. truncatus* stranded in Lanzarote, Canary Islands, in 2005 [69]. Thus, at least two strains of CeMV are circulating in cetaceans from this ocean province.

#### 6.1.2. Mediterranean Sea

In the Mediterranean Sea, DMV caused two well-documented outbreaks of mass mortality in *S. coeruleoalba* in 1990–1992 and in 2006–2008. The first outbreak started in Valencia, Spain, in July 1990 and extended to France, Italy, Greece and Morocco, ending in the spring of 1992. All age classes were affected but most dead dolphins were adults. Although precise mortality rates could not be determined, thousands of animals are thought to have died [127,137]. As an indirect measure of the impact, the mean school size in the areas most affected by the 1990–1992 outbreak significantly decreased to less than 30% of the pre-outbreak numbers [127,137]. Serological surveys carried out during and after the epidemic indicated that in 1997–1999 only adult dolphins had DMV antibodies and that the prevalence of seropositivity in mature dolphins had decreased from 100% (*N* = 8) in 1990–1992 to 50% (*N* = 6) in 1997–1999, but sample sizes were small [63]. This suggested that DMV had not persisted in *S. coeruleoalba* after the epidemic ended, presumably because their abundance (117,880 CI = 68,379–148,000) in the western Mediterranean Sea [137] was too low to support endemic infection [63,67]. Histological and IHC surveillance further supported this hypothesis. Indeed, systemic CeMV was not detected in 50 *S. coeruleoalba* stranded along the Catalonian coast in the inter-epidemic period [56]. Between October 2006 and April 2007, at least 27 morbillivirus-infected *G. melas* stranded along the southern Spanish Mediterranean coast and the Balearic Islands. The outbreak was first recorded in the Strait of Gibraltar area in late October 2006 and then spread to Valencia [67,88]. In early July 2007 DMV-infected *S. coeruleoalba* were observed in the Gulf of Valencia [67]. The number of animals washed ashore from July through August 2007 was similar to that recorded in 1990 during the same months. The stranding rate was also similar during each episode, with an initial low rate at the beginning of July and then a sharp increase in mid-August [67]. The outbreak extended to France and Italy during the following months, also affecting *T. truncatus* [32,138]. Mostly juveniles were affected during this mortality event, likely because adults were still protected by immunity acquired during the 1990–1992 epidemic [32,67]. The virus strains amplified by RT-PCR from tissues of *S. coeruleoabalba*, *G. melas* and *T. truncatus* were similar to those isolated during the 1990–1992 epidemic but not identical ([27,31,32,67,88], this paper). An estimated 200 striped dolphins died in the western Mediterranean but the total number of deaths remains unknown [67]. As well as the deaths caused by the acute infection, there were also several cases, ultimately lethal, of a chronic CNS form of infection in 1991–1994 and 2008–2011 in the western Mediterranean and in 2009–2011 in the Eastern Mediterranean [56,66,94]. In the Western Mediterranean chronic morbillivirus encephalitis represented the most common single cause of stranding and death in mature *S. coeruleoalba* in the years following a DMV epizootic [56]. These data suggest that the second DMV outbreak may also have had a negative impact on the Mediterranean *S. coeruleoalba* population, though to a lesser extent than the previous one. Little is known about the impacts of the outbreak on populations of the other cetacean species affected. However, Wierucka *et al*. [139] found that the 2006–2008 DMV epidemic lowered the survival rate of some clusters (groups of individuals that associate with each other more often than with others) of *G. melas* (from 0.919 (95% CI: 0.854−0.956) to 0.547 (95% CI: 0.185−0.866)) in the Alboran Sea and Gulf of Vera.

The *S. coeruleoalba* population density in the Gulf of Valencia (0.49 dolphin/km^2^) was again close to the maximum reported for this species in the Western Mediterranean in 2001–2003 [104,140]. This high population density, with a large proportion of susceptible individuals, likely favored viral transmission and permitted the start of a new epidemic when DMV was reintroduced into the population [67]. As both the 1990–1992 and 2006–2007 DMV epidemics started close to, or in, the Gibraltar Strait, it was suggested that DMV endemically infected cetaceans, possibly *G. melas* transmitted the infection to *S. coeruleoalba* with which they occasionally form mixed groups (Raga *et al*. pers. observations). The recent detection of DMV strains in *S. coeruleoalba* from the CE Atlantic Ocean that are almost identical to the Mediterranean strains [33] indicates that this population could also transmit the virus to the Mediterranean *S. coeruleoalba* through occasional contacts in the Strait of Gibraltar. The finding of systemic morbillivirus infection in two adult *S. coeruleoalba* stranded on the southwestern (Atlantic) coast of Spain, close to Gibraltar in 2011 and 2012 [141] further indicates that this Strait plays an important role in the epidemiology of CeMV. Environmental factors (higher sea-surface temperatures and limited prey availability), as well as fisheries interactions, inbreeding, migration, and high contaminant loads may synergistically interact to increase the severity of the disease and favor transmission between species [127,142,143,144,145]. When CeMV herd immunity significantly decreases in Mediterranean *S. coeuruleoalba,* the population will again be at risk for an epidemic. Serological surveys are needed to determine the current immune status of these dolphins and facilitate development of predictive epidemiological models. 

Recently, morbilliviral RNA was detected by RT-PCR in brain and lung samples from 22 of 52 *S. coeruleoalba*, one of three *T. truncatus* and one *B. physalus* stranded along the Italian Tyrrhenian Sea coast during an unusual mortality event in early 2013 ([146], Di Guardo and Mazzariol, pers. observations). However, as none of the positive individuals had characteristic morbillivirus lesions and, as other infectious agents were concurrently detected in a high percentage of these individuals, the proximate cause of the event is still under investigation (Di Guardo and Mazzariol, pers. observations.).

#### 6.1.3. Black Sea

Two *D. delphis ponticus* that stranded during an outbreak of mortality in Crimea in August and September 1994 had broncho-pneumonia, syncytia and lymphoid depletion [117]. Morbillivirus antigen was observed by IHC in the lungs, cerebrum, spleen and lymph nodes. However, morbillivirus RNA could only be detected in a formalin-fixed sample of a testicular fibroma by RT-PCR. There was no evidence of morbillivirus in the frozen tissues using either virus isolation or an antigen capture ELISA that had proven useful during other mortality events [3,22,117]. As virus isolation was negative and sequencing of the PCR products was not performed, it is unclear which morbillivirus caused the death of these dolphins. The last *S. coeruleoalba* reported to die of acute DMV infection in Greek waters was found in the spring of 1992, two years before the *D. delphis ponticus* mortality in the Black Sea. *S. coeruleoalba* are not known to enter the Turkish Strait Systems (Bosphorus, Marmara Sea and Dardanelles) where *D. delphis* *ponticus* are commonly seen, and are absent from the Black Sea [147]. *D. delphis ponticus* have also not been reported in Aegean waters [147]. Thus, a link between the 1990–1992 morbillivirus outbreak in Mediterranean *S. coeruleoalba* and the morbillivirus infection in the two *D. delphis ponticus* stranded in Crimea is unclear. Whether the morbillivirus was in fact CeMV, originating in the Mediterranean, or another morbillivirus should be further examined.

### 6.2. North America

There have been several die-offs in coastal *T. truncatus* populations from the Gulf of Mexico and the Atlantic coast of the US since 1982 [25,57,80,92,130,148]. 

#### 6.2.1. Atlantic Coast

From January to May of 1982, 43 carcasses were recovered in the Indian River Lagoon System (IRL), Florida, among a community estimated at 211 individuals [129]. Serological data indicated that this outbreak was likely due to a morbillivirus infection and contact with endemically infected species such as offshore *T. truncatus* was hypothesized to be the source of infection for the event [25]. Further serological studies performed on samples collected from 2003–2007 indicated that IRL dolphins born after the 1982 mortality had antibodies to a DMV-like virus, indicating exposure and infection, though no outbreaks or associated deaths were documented after 1982 [64]. 

From June 1987 to May of 1988, CeMV infected both inshore and estuarine *T. truncatus*, starting from New Jersey and, eventually, reaching Florida [25,57,92,148]. This mass mortality was associated with the stranding of at least 645 *T. truncatus* [148]. DMV infection was also detected in a *S. coeruleoalba*, indicating that multiple species were affected during that outbreak [92]. Starting in July 2013, another outbreak affected these populations along the Atlantic coast from New York through northern Florida and is currently ongoing [71]. Over 1500 *T. truncatus* have died and the majority of the individuals tested by RT-PCR have been positive for DMV [71,130]. Contacts between inshore *T. truncatus* and offshore species (offshore *T. truncatus*, *Globicephala* sp., *S. coeruleoalba*, *L. hosei*, and false killer whales (*Pseudorca crassidens*)) in which CeMV is endemic [23,24,25] may have been the sources of infection for the 1987–88 and 2013–2014 outbreaks. Seasonal overlap between resident coastal *T. truncatus* stocks at certain times of the year, and migration of the coastal migratory stock, may have favored transmission of the disease down the coast [149]. A serological survey performed on samples collected from live capture-released coastal and estuarine *T. truncatus* along the east coast of the US in 1999–2004 indicated that the seroprevalence decreased over the years of the study, suggesting that CeMV did not persist as an endemic infection in these populations, as had been predicted [25,26]. Therefore, population immunity likely continued to decrease over time leading to increased numbers of susceptible individuals and resulting in the 2013–2014 epidemic. The role of environmental and anthropogenic factors in this mortality and the population impacts is being investigated [71].

#### 6.2.2. Gulf of Mexico

In 1993–1994, CeMV caused another outbreak of mortality, this time in a population of *T. truncatus* from the Gulf of Mexico, spanning from Florida (Panama City) to Texas [92,150]. A total of 171 specimens were retrieved from the entire Texas coast in March and April 1994 [151]. About a quarter of 34 dolphins sampled in Matagorda Bay in 1992 had CeMV antibodies, indicating that this community/population had been exposed to the virus before the 1993–1994 outbreak occurred [25].

#### 6.2.3. North Pacific

Morbillivirus infection was detected by RT-PCR in a juvenile male *I. pacificus* stranded at Hana, Maui in March 2010, following traumatic maxillary and mandibular bone fractures [5]. The whale had chronic encephalitis and was also concurrently infected by an alphaherpesvirus [5]. Though morbillivirus RNA was detected by RT-PCR in samples of the lungs, spleen, thymus, and lymph nodes the juvenile did not have any typical morbillivirus lesions in these organs [5]. This may reflect prolonged persistence of morbilliviral RNA following acute infection, as described for MV [93] but further analyses are necessary to confirm this hypothesis. The virus represents a new strain of CeMV-1, tentatively named beaked whale morbillivirus. BWMV was also detected by RT-PCR in the tracheobronchial lymph node and spleen of a neonate *P. macrocephalus* beached on the island of Oahu, Hawaii, in May 2011. However, typical morbillivirus lesions were not detected in this individual [121]. 

Six of 18 *D. delphis* that stranded along the coast of California from August 1995 through August 1997 had serum antibodies against DMV. Morbilliviral RNA was detected in the normal spleen and heart of two seropositive dolphins that did not show any typical morbillivirus lesions and in the brain of a third dolphin that suffered mild lymphocytic meningoencephalitis [75]. Together, these data indicate that CeMV strains are circulating in the North Pacific. 

### 6.3. South America

Although CeMV infection had already been detected by serology in gregarious odontocetes from Peru, Argentina and Brazil in the late 1990s ([62,63]; Table 1), morbillivirus mortalities were not observed until recently [6]. A new lineage of CeMV was detected in an emaciated *S. guianensis* calf washed ashore dead at Guriri, Espírito Santo State, Brazil, in November 2010 with marked lymphoid depletion, interstitial pneumonia, and meningoencephalitis [6]. The *S. guianensis* community off Guriri may be related to the Abrolhos Bank population that concentrates around the estuaries of Caravelas (estimated at 57–124 individuals) and Doce rivers [152,153]. Though *S. guianensis* have not been observed mixing with other cetacean species in this region, it is sympatric with the rough-toothed dolphin (*Steno bredanensis*), *T. truncatus*, the humpback whale (*Megaptera novaeangliae*) and the southern right whale (*Eubalaena australis*) [154,155]. Interactions between these species may have resulted in the infection of the *S. guianensis* calf. Preliminary IHC studies suggested that morbilliviruses have infected other cetacean species along the Brazilian coast [156]. 

### 6.4. Asia and Australasia

#### 6.4.1. Asia

CeMV infection was detected by serology, IHC and RT-PCR in a stranded *L. obliquidens* from Japan, in a pygmy sperm whale (*Kogia breviceps*) beached in Taiwan and in a captive *T. aduncus* from Taiwan [63,70,106]. The *L. obliquens* was diagnosed with a chronic persistent morbillivirus encephalitis while the *K. breviceps* had a systemic, acute infection [70,106]. Partial sequence of the P gene of the *K. breviceps* virus had 97.6% similarity with DMV. The *T. aduncus* had very high titers against DMV likely acquired after an infection developed while still in the wild [63]. Though these data indicate that CeMV is present in odontocetes from the Northwest Pacific, mass mortalities were not reported in this ocean basin. Further investigations are necessary to determine the distribution of virus, the identity of strains and susceptibility of hosts in this region. 

#### 6.4.2. Australasia

Serological data indicate that CeMV infects several cetacean species from the waters of northeastern Australia, Tasmania and New Zealand and that it is possibly endemic in *G. melas* and *P. electra*, with evidence of circulation in this region as long ago as 1985 [63,90]. On Australia’s Indian Ocean coast, CeMV infection was diagnosed in two *T. aduncus* from the Swan River, Western Australia that died during an unusual mortality event involving a small resident community of ~25 individuals in June 2009 [7]. These deaths were soon followed by morbillivirus associated mortalities in two immature offshore *T. truncatus* in Queensland, Eastern Australia [68,90]. One was found dead on North Stradbroke Island in 2009, the other beached on Fraser Island in 2010 [68,90]. The Queensland *T. truncatus* were infected by a virus closely related to the DMV strain isolated in Europe and the USA and exhibited classical acute infection [68,90]. However, the Western Australia *T. aduncus* had an unusual form of CeMV characterized by severe lymphoid depletion and massive opportunistic infections and were infected by a strain of CeMV-2 ([7], this paper). Another morbillivirus outbreak was subsequently reported in *T. aduncus* and *T. truncatus* from South Australia in March-September 2013 [51,131]. Preliminary sequencing data suggest that the Western Australia and South Australia morbilliviruses are more similar to each other than either are to the Queensland strain (J. Wang, pers. observations). Together these data suggest that CeMV-1 and CeMV-2 are widespread in the waters of Australasia and could cause more mortalities in inshore and estuarine dolphins, in addition to offshore dolphins. 

## 7. Conclusions 

Significant progress in our understanding of the epidemiology, molecular biology and pathogenesis of CeMV have been made since PMV and DMV were first detected in small odontocetes in European waters in 1988–1992. Large herds of gregarious species were found to be the likely reservoirs and sources of CeMV infection to susceptible species in the Atlantic and Pacific Oceans [23,24,25,61,62,63,90]. New species and lineages of CeMV have been recently discovered [5,6,7]. Several techniques have been developed to optimize the diagnosis of CeMV infection, to differentiate the strains and to reduce the possibility of cross-contamination [16,20,55]. Serological assessment may enable prediction of future outbreaks [157]. The development of Next Generation Sequencing technologies has greatly enhanced the detection and genetic characterization across all forms of life [158]. To date, such technologies have not yet been applied to morbillivirus infections of aquatic mammals although they recently enabled an assessment of the evolution of ruminant morbilliviruses [159] and their application to cetacean morbilliviruses may allow a greater understanding of their evolution. Such studies may, where sampling permits, enable the use of genetic data to trace transmission routes between cetacean species and indicate key interactions between species that could lead to significant outbreak events. Standard sampling and preservation protocols should be used during suspected morbillivirus outbreaks and complete genomes of CeMV strains and lineages should be sequenced [19]. The recent discoveries of several new morbilli-related viruses in bats [160], as well a potentially novel feline morbillivirus representing a basal divergence in the genus [11], are likely to lead to a revision of the phylogeny and understanding of the evolution of morbilliviruses. Identification of the SLAM cell receptor in several cetacean species [9,13] represents a major step in our understanding of the pathogenesis of CeMV infection, especially with regard to susceptibility and transmission to non-classical hosts, such as pinnipeds [9,13,161,162]. Further studies should confirm whether the SLAM cell receptor is indeed the primary immune receptor for CeMV, as is the case for other studied morbilliviruses, and should look for the nectin4 epithelial cell receptor and other cell receptors in cetaceans. Further studies are also warranted to delineate the host responses to CeMV strains and lineages, and the factors that determine the outcome of infection in cetaceans. Mathematical models should be developed to examine the long-term dynamic consequences of the epidemics on odontocete populations and to predict the risk of epidemics, as has been done for PDV in harbor seals (*Phoca vitulina*) [157,163]. The concurrent use of the different diagnostic techniques in the context of an integrative approach that includes epidemiological parameters, life history of the affected species and environmental parameters should provide a better and more complete picture of the ecology and evolution of CeMV.
